# *Doenjang* (Traditional Korean Fermented Soy Paste) Attenuates Development of Colitis-Associated Colorectal Cancer by Modulating Apoptotic, Inflammatory, and Gut Microbiota Pathways

**DOI:** 10.3390/foods14203565

**Published:** 2025-10-20

**Authors:** Hyeon-Ji Lim, In-Sun Park, Min Ju Kim, Ji Won Seo, Gwangsu Ha, Hee-Jong Yang, Do-Youn Jeong, Seon-Young Kim, Chan-Hun Jung

**Affiliations:** 1 Jeonju AgroBio-Materials Institute, Wonjangdong-gil 111-27, Jeonju 54810, Republic of Korea; lhj0923@jami.re.kr (H.-J.L.); witwit58@jami.re.kr (I.-S.P.); mjkim92@jami.re.kr (M.J.K.); seon02@jami.re.kr (S.-Y.K.); 2 Microbial Institute for Fermentation Industry, Sunchang 56048, Republic of Korea; wldnjs8769@naver.com (J.W.S.); ksnova1492@naver.com (G.H.); godfiltss@naver.com (H.-J.Y.); jdy2534@korea.kr (D.-Y.J.)

**Keywords:** *Doenjang*, colorectal cancer, inflammation, apoptosis, gut microbiota

## Abstract

Colitis-associated colorectal cancer (CAC) is a type of colorectal cancer (CRC) that develops as a result of chronic inflammation, particularly in patients with inflammatory bowel diseases such as ulcerative colitis and Crohn’s disease. Persistent intestinal inflammation and dysbiosis of gut microbiota under these conditions are major contributors to CRC progression. *Doenjang*, a traditional Korean fermented soybean paste, contains probiotics that influence intestinal microbiota composition, as well as biogenic amines (BAs), harmful compounds generated during fermentation. We analyzed the bacterial composition and BA content of *Doenjang* and evaluated its effects on CAC in a mouse model of AOM/DSS-induced CAC. Results revealed that *Doenjang* contains diverse beneficial probiotics alongside BAs. *Doenjang* significantly reduced tumor formation and attenuated CAC progression by modulating inflammatory and apoptotic pathways and suppressed the expression of pro-inflammatory cytokines (TNF-α, IL-1β, and IL-6) by inhibiting the NF-κB pathway. Additionally, *Doenjang* restored intestinal epithelial barrier integrity by increasing the expression of mucin-related genes (MUC-2, MUC-3) and protective factors like TFF-3. Gut microbiota analysis revealed that *Doenjang* promoted a healthier gut environment by increasing microbial diversity and reducing inflammation-related bacterial populations. These findings suggest that *Doenjang* helps prevent and manage CAC by modulating inflammatory responses and gut microbiota composition.

## 1. Introduction

Colorectal cancer (CRC) is one of the most common malignant tumors worldwide, with its incidence steadily increasing, particularly in Asian populations due to the adoption of Westernized diets [1]. Current treatments, including surgery, radiation therapy, immunotherapy, and chemotherapy, are widely used in clinical practice to manage CRC [2]. However, these approaches often lead to severe side effects and drug resistance, resulting in decreased survival rates and compromised quality of life [3]. Therefore, there is a growing need for new, safe, and effective therapeutic approaches to prevent CRC progression and improve intestinal health.

The exact mechanisms of CRC development remain unclear, but chronic inflammation has been identified as a critical driver of its progression [4,5]. In addition, the gut microbiome is highly correlated with CRC development [6]. The NF-κB signaling pathway plays a key role in promoting pro-inflammatory cytokines, such as TNF-α, IL-1β, and IL-6, which are involved in tumor initiation and progression [7]. Persistent NF-κB activation has also been linked to gut microbiota dysbiosis, which disrupts microbial homeostasis, contributing to chronic intestinal inflammation and promoting colitis-associated carcinogenesis [8].

In addition to inflammation, disruptions in apoptotic pathways play a key role in CRC progression [9]. Apoptosis, or programmed cell death, is essential for eliminating damaged or abnormal cells. In CRC, apoptosis is often dysregulated, allowing cells to evade death and continue proliferating [9,10]. The apoptotic process is tightly regulated by the balance between pro-apoptotic proteins, such as BAX, and anti-apoptotic proteins, such as BCL-2 and BCL-xL [11]. Overexpression of anti-apoptotic proteins is associated with tumor progression and poor prognosis, making apoptotic regulation a promising target for cancer therapy [11,12].

Fermented foods, such as *Doenjang* [13], cheonggukjang [14], kimchi [15], natto [16], and miso [17], are known for their anti-inflammatory and anticancer properties. *Doenjang*, a traditional Korean fermented soybean paste, is produced by fermenting Meju (soybean blocks) with various microorganisms, including *Bacillus* species, lactic acid bacteria, and fungi [13]. *Doenjang* is usually produced using either traditional or commercial methods, with traditional methods typically fermenting using regionally specific microorganisms [18]. These microorganisms play a crucial role in breaking down complex soy proteins into bioactive peptides and producing various metabolites, such as flavonoids, phenolic acids, and isoflavones [13,19]. However, during fermentation, harmful substances, such as biogenic amines (BAs), can be generated [19].

In this study, we investigated the BA content of *Doenjang* samples manufactured using traditional and commercial methods and evaluated their functional differences using a mouse model of azoxymethane/dextran sulfate sodium (AOM/DSS)-induced colitis-associated colorectal cancer (CAC).

## 2. Materials and Methods

### 2.1. Information on Doenjang

The four *Doenjang* samples used in this study were provided by the Microbial Institute for Fermentation Industry (MIFI, Sunchang, Jeonbuk, Republic of Korea). Detailed information on the samples is presented in Table 1. All samples were diluted in distilled water and administered orally to mice at a concentration of 200 mg/kg, based on previous studies using similar fermented foods [14,20].

### 2.2. Analysis of Biogenic Amines and Sodium Content

BAs (histamine and tyramine) in fermented soybean products were quantified according to the Korean Food Code, as established by the Ministry of Food and Drug Safety [21]. Approximately 5 g sample was homogenized with 25 mL of 0.1 N HCl and centrifuged at 4500 rpm for 15 min at 4 °C. The supernatant was collected, and the residue was re-extracted under identical conditions. The combined supernatants were adjusted to a final volume of 50 mL. An aliquot (0.5 mL) of the extract was mixed with 0.25 mL internal standard solution (1,7-diaminoheptane, 0.1 g/L), 0.25 mL saturated sodium carbonate, and 0.4 mL of 1% dansyl chloride. The mixture was incubated at 45 °C for 1 h to allow derivatization. Residual dansyl chloride was quenched by the addition of 0.25 mL of 10% proline solution. The derivatized sample was extracted with 2.5 mL of ethyl ether by vigorous shaking for 3 min, and the organic phase was subsequently collected. The solvent was evaporated to dryness under a nitrogen stream, and the residue was reconstituted in 0.5 mL of acetonitrile. The resulting solution was passed through a 0.45-μm syringe filter and analyzed by HPLC.

Sodium content was quantified using inductively coupled plasma–mass spectrometry (ICP–MS). Samples were digested with concentrated acid using a microwave digestion system, diluted to a sodium concentration of 1–10 µg/mL, and analyzed by ICP–MS.

### 2.3. Microbial Analysis

Bacterial DNA was extracted using the PowerFood Microbial Kit (Qiagen, Hilden, Germany) from four selected *Doenjang* samples. The extracted DNA was then used to construct a 16S rRNA gene library targeting the V3–V4 regions and sequenced using the Illumina MiSeq platform. The resulting FASTQ files were processed using EzBioCloud 16S-based microbiome taxonomic profiling (MTP, Chunlab Inc., Seoul, Republic of Korea). During the analysis, chimeric, low-quality, and non-bacterial sequences were filtered out, and the remaining sequences were clustered into operational taxonomic units (OTUs) based on a 97% similarity threshold using the EzTaxon database PKSSU (v.4.0). Taxonomic classification was conducted at various levels, including phylum and genus. The microbial diversity within each sample was evaluated by calculating α-diversity indices, including Chao1 and Shannon indices, using the MTP browser tool. To validate the statistical robustness of the sequencing results, Good’s coverage analysis was also performed, confirming that obtained number of reads was sufficient for reliable microbiome analysis.

### 2.4. AOM/DSS-Induced CAC Study

Four-week-old male specific pathogen-free (SPF) BALB/c mice (*n* = 35, 18–20 g) were purchased from Damool Science (Daejeon, Republic of Korea) and acclimated for one week under controlled environmental conditions (22 ± 2 °C, 55 ± 5% humidity, 12 h light/dark cycle). During acclimation, all animals had free access to standard chow diet and water. Following acclimation, mice were randomly allocated into seven groups (*n* = 5 per group) based on their average body weight: Normal (N), Negative Control (AOM/DSS + C), Positive Control (AOM/DSS + PC; 5-aminosalicylic acid 75 mg/kg), and four *Doenjang*-treated groups (AOM/DSS + DJA, DJB, DJC, DJD; 200 mg/kg each), yielding a total of 35 mice analyzed. To minimize social interaction across groups, animals belonging to the same experimental group were housed together within the same cage. The sample size (five per group) was based on previous studies employing similar fermented foods [14,20]. As shown in Table 2, the *Doenjang* sample groups and PC group were pre-treated orally with traditional fermented soybean paste products and 5-aminosalicylic acid (5-ASA; Sigma-Aldrich, St. Louis, MO, USA) for eight days, respectively. Thereafter, the respective treatments were continued by oral gavage five days per week until the end of the study, resulting in a total oral administration duration of fifty days. After pre-treatment, azoxymethane (AOM; Sigma) at 10 mg/kg was injected intraperitoneally to induce colon carcinogenesis. After one week, 2% dextran sodium sulfate (DSS; MP Biomedicals, Irvine, CA, USA) was provided in the drinking water supplied to the mice for seven days, followed by a two-week recovery period with regular water. This DSS cycle was repeated once more, resulting in a total experimental period of eight weeks. Animals were monitored two times per week for body weight and signs of distress. Fecal samples were collected 24 h before the end of the experimental period and stored at −80 °C until analysis. At the end of the experiment, colon tissues and serum samples were collected and stored at −80 °C for further analysis. The animal experiments and data analysis were performed independently by separate researchers. No animals or data points were excluded from the experiments, and all animals and measured data points were included in the final analysis. All animal experiments were approved by the Jeonju AgroBio-Materials Institute Animal Care and Use Committee (Approval No. JAMI IACUC 2024004; Approval Date 30 April 2024).

### 2.5. Enzyme-Linked Immunosorbent Assay (ELISA)

For cytokine analysis, blood samples were collected at the end of the experimental period and allowed to coagulate at room temperature for 30 min. The samples were then centrifuged at 12,000 rpm for 15 min to separate the serum. The separated serum was stored at −80 °C until further analysis. Serum levels of tumor necrosis factor-alpha (TNF-α), interleukin-1 beta (IL-1β), and interleukin-6 (IL-6) were measured using ELISA kits according to the manufacturer’s instructions (R&D Systems, Minneapolis, MN, USA). Absorbance was measured at 450 nm using a microplate reader (Thermo Fisher Scientific, Wilmington, MA, USA), and cytokine concentrations were calculated based on a standard curve generated using recombinant cytokines provided in the assay kit.

### 2.6. Quantitative Real-Time PCR (qRT-PCR) Analysis

Total RNA was extracted from colonic tissues using a Hybrid-R kit (GeneAll, Seoul, Republic of Korea). cDNA was synthesized using the 2× RT Pre-Mix (BioFACT, Daejeon, Republic of Korea), followed by qRT-PCR amplification using the 2× Real-Time PCR Master Mix (BioFACT). The reactions were analyzed using a CFX96 sequence detection system (Bio-Rad, Hercules, CA, USA). The primer sequences used for the target genes are listed in Table 3.

### 2.7. Western Blotting

Colon tissues were homogenized and lysed using ice-cold RIPA buffer (Thermo Fisher Scientific) containing protease and phosphatase inhibitor cocktails (GenDepot, Barker, TX, USA). The lysates were separated by centrifugation at 13,000 rpm for 20 min at 4 °C, and the supernatant was collected. Protein concentration was determined using the BCA Protein Assay Kit (Bio-Rad, Hercules, CA, USA). The membranes were blocked with 5% skim milk in Tris-buffered saline with 0.1% Tween-20 (TBST) for 1 h at room temperature and then incubated overnight at with primary antibodies against iNOS, COX-2, phospo-p65, p65, phospo-IkB, IkB, Bax, p53, Bcl-2, Bcl-XL, and β-actin (Cell Signaling Technology, Danvers, MA, USA) at 4 °C. After washing, the membranes were treated with an enhanced chemiluminescence (ECL) reagent (GE Healthcare, Chicago, IL, USA) and visualized using an Amersham Imager 600 gel imaging system (GE Healthcare).

### 2.8. Hematoxylin and Eosin (H&E), Alcian Blue (AB), and Immunohistochemical (IHC) Staining

Colon tissues were fixed in 10% formalin, embedded in paraffin, and sliced into 4-µm sections. The sections were deparaffinized with xylene, rehydrated through ethanol washes, and rinsed with water. For H&E staining, sections were stained with hematoxylin and eosin and dehydrated. For AB staining, sections were incubated with 1% AB (pH 2.5) for 30 min, rinsed, counterstained with nuclear fast red, dehydrated, and mounted. For IHC, sections were treated with 0.3% H_2_O_2_, subjected to antigen retrieval in citrate buffer (pH 6.0), and were blocked with 4% BSA. Then, samples were probed overnight with primary antibodies, including anti-PCNA (Cell Signaling Technology), anti-MUC-2, and anti-TFF-3 (Abcam, Cambridge, UK), at 4 °C. This was followed by incubation with HRP-conjugated secondary antibodies and hematoxylin counterstaining. All stained sections were analyzed using a digital slide scanner (Motic, Xiamen, China).

### 2.9. Statistical Analysis

Statistical comparisons between groups were performed using one-way ANOVA, followed by Tukey’s post hoc test in GraphPad Prism 5 (San Diego, CA, USA). Data distribution normality was assessed using the Kolmogorov–Smirnov test, and all groups met the assumption of normality (*p* > 0.05). Although a formal test for homogeneity of variances (e.g., Levene’s test) was not available in this software version, visual inspection of variance and residual plots showed comparable group variances. Thus, parametric analysis was deemed appropriate. Results are expressed as mean ± standard deviation (SD), with *p* < 0.05 considered statistically significant.

## 3. Results

### 3.1. Bacterial Composition, BAs, and Salt Contents in Doenjang

**Figure 1:**
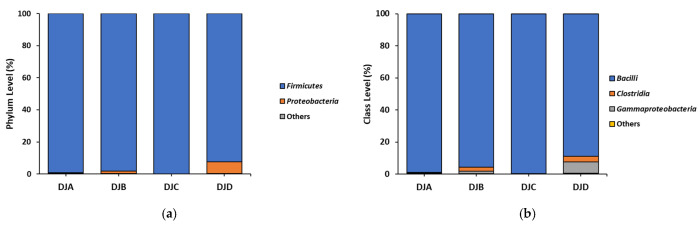

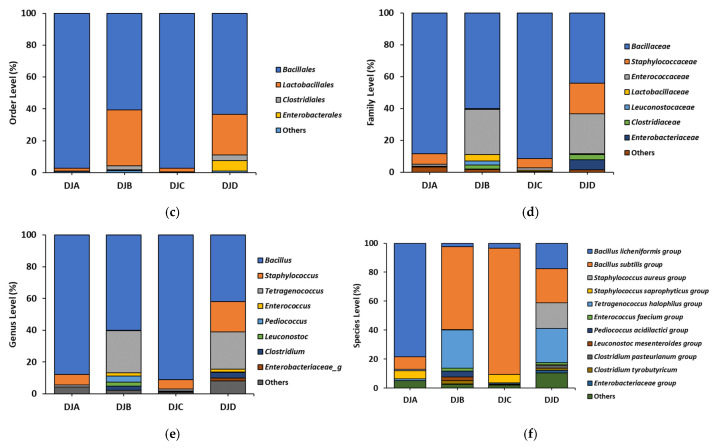
Microbial community composition in *Doenjang* at the (**a**) phylum, (**b**) class, (**c**) order, (**d**) family, (**e**) genus, and (**f**) species levels assessed using next-generation sequencing analysis.

To analyze the bacterial composition of *Doenjang*, Next-Generation Sequencing was performed. The four *Doenjang* samples revealed a predominance of Firmicutes and Bacilli at the phylum and class level, respectively (Figure 1a,b). At the order level, Bacillales was the most predominant (≥90%) in DJA and DJC. Bacillales (≥60%) and Lactobacillales (≥25%) were sequentially dominant in DJB and DJD (Figure 1c). At the family level, Bacillaceae was the most predominant (≥80%) in DJA and DJC. Bacillaceae (≥40%) and Enterococcaceae (≥25%) were sequentially dominant in DJB and DJD (Figure 1d). At the genus level, *Bacillus* was the most predominant (≥80%) in DJA and DJC. *Bacillus* (≥40%) and *Tetragenococcus* (≥20%) were sequentially dominant in DJB and DJD (Figure 1e). At the species level, *Bacillus licheniformis* (78.51%) was the highest in the DJA, followed by that of *Bacillus subtilis* (8.61%) and *Staphylococcus saprophyticus* (5.70%). In DJB, *Bacillus subtilis* (57.44%) and *Tetragenococcus halophilus* (26.39%) were sequentially dominant. In DJC, *Bacillus subtilis* (87.01%) was most predominant, whereas DJD was dominated by *Bacillus subtilis* (23.55%), *Tetragenococcus halophilus* (23.54%), *Staphylococcus aureus* (17.70%), and *Bacillus licheniformis* (17.56%) (Figure 1f).

To analyze the level of the BAs—histamine and tyramine—in *Doenjang*, HPLC was performed. The histamine content was 141.9 mg/kg in DJA, 151.13 mg/kg in DJB, 794.86 mg/kg in DJC, and 928.61 mg/kg in DJD. On the other hand, the tyramine content was 284.27 mg/kg, 886.86 mg/kg, 215.89 mg/kg, and 186.59 mg/kg in DJA, DJB, DJC, and DJD, respectively (Table 1).

### 3.2. Doenjang Reduces CAC-Associated Features in Mice with AOM/DSS-Induced CAC

**Figure 2:**
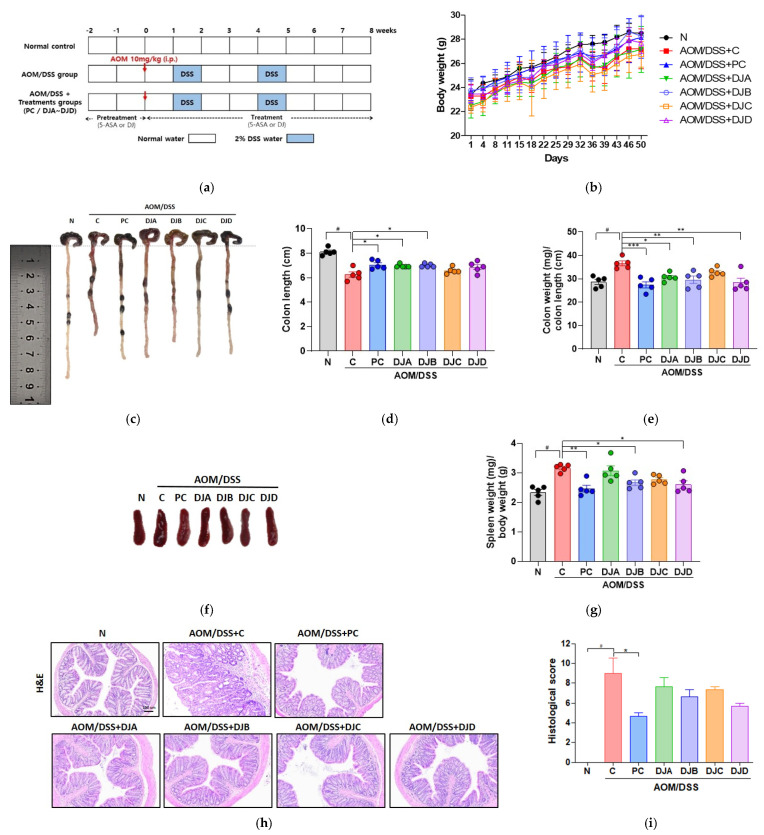
*Doenjang* attenuates CAC-associated features in mice with AOM/DSS-induced CAC. (**a**) Experimental schedule. (**b**) Body weight changes during the experimental period. (**c**) Representative images of colons from each group. (**d**) Colon length was measured at the end of the experiment. (**e**) Colon weight-to-length ratio (g/cm). (**f**) Representative images of spleens from each group. (**g**) Spleen weight relative to the body weight (mg/g). (**h**,**i**) Histopathological evaluation of colon tissue by H&E staining and corresponding histological scores. N, Normal control group; C, AOM/DSS group; PC, 75 mg/kg 5-ASA + AOM/DSS; DJA–DJD, *Doenjang* sample groups + AOM/DSS. # *p* < 0.05, compared to the N group; * *p* < 0.05, ** *p* < 0.01, and *** *p* < 0.001, compared to the C group.

To evaluate the effects of *Doenjang* on CAC progression, body weight, colon length, colon weight-to-length ratio, and spleen weight were measured in mice with AOM/DSS-induced CAC. Body weight changes were monitored over the course of the experiment, with no significant differences observed between groups (Figure 2b). Colon length, a marker of colonic inflammation, was significantly reduced in the C group (6.3 ± 0.2 cm) compared to the N group (8.1 ± 0.3 cm). Treatment with 5-ASA (PC group, 8.1 ± 0.3 cm) and *Doenjang* samples restored colon length, with significant improvements in the DJA (7.1 ± 0.4 cm) and DJB (7.0 ± 0.1 cm) groups (Figure 2c,d). Colon weight relative to colon length (mg/cm) was measured to assess pathological changes in colonic tissue, including inflammation and tumor development (Figure 2e). The C group (36.6 ± 2.3 mg/cm) showed a significantly higher ratio compared to the N group (28.7 ± 2.3 mg/cm), indicating severe colonic inflammation and tumor progression. In contrast, treatment with 5-ASA (PC group, 27.4 ± 2.9 mg/cm) and *Doenjang* samples reduced this ratio, with significant reductions observed in the DJA (30.7 ± 1.8 mg/cm), DJB (29.6 ± 3.4 mg/cm), and DJD (28.5 ± 4.0 mg/cm) groups. Spleen weight, an indicator of immune system activation, was significantly increased in the C group (3.2 ± 0.1 mg/g) compared to the N group (2.3 ± 0.2 mg/g). Treatment with 5-ASA (PC group, 2.5 ± 0.3 mg/g) and *Doenjang* samples, particularly DJB (2.6 ± 0.1 mg/g) and DJD (2.6 ± 0.3 mg/g), significantly reduced spleen weight, indicating modulation of the immune responses (Figure 2f,g). Colon tissue integrity was evaluated by H&E staining (Figure 2h,i). The C group showed severe epithelial damage and crypt loss, whereas PC group markedly preserved mucosal architecture. *Doenjang*-treated groups exhibited partial epithelial protection, indicating a trend of improvement although not statistically significant. These results suggest that *Doenjang* treatment can protect against AOM/DSS-induced colon injury.

### 3.3. Doenjang Suppresses Tumor Growth by Regulating Apoptosis and Proliferation in CAC Mice

**Figure 3:**
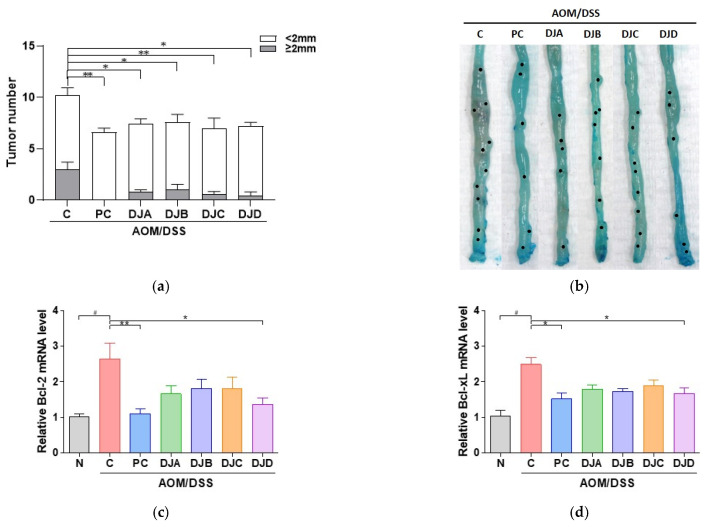

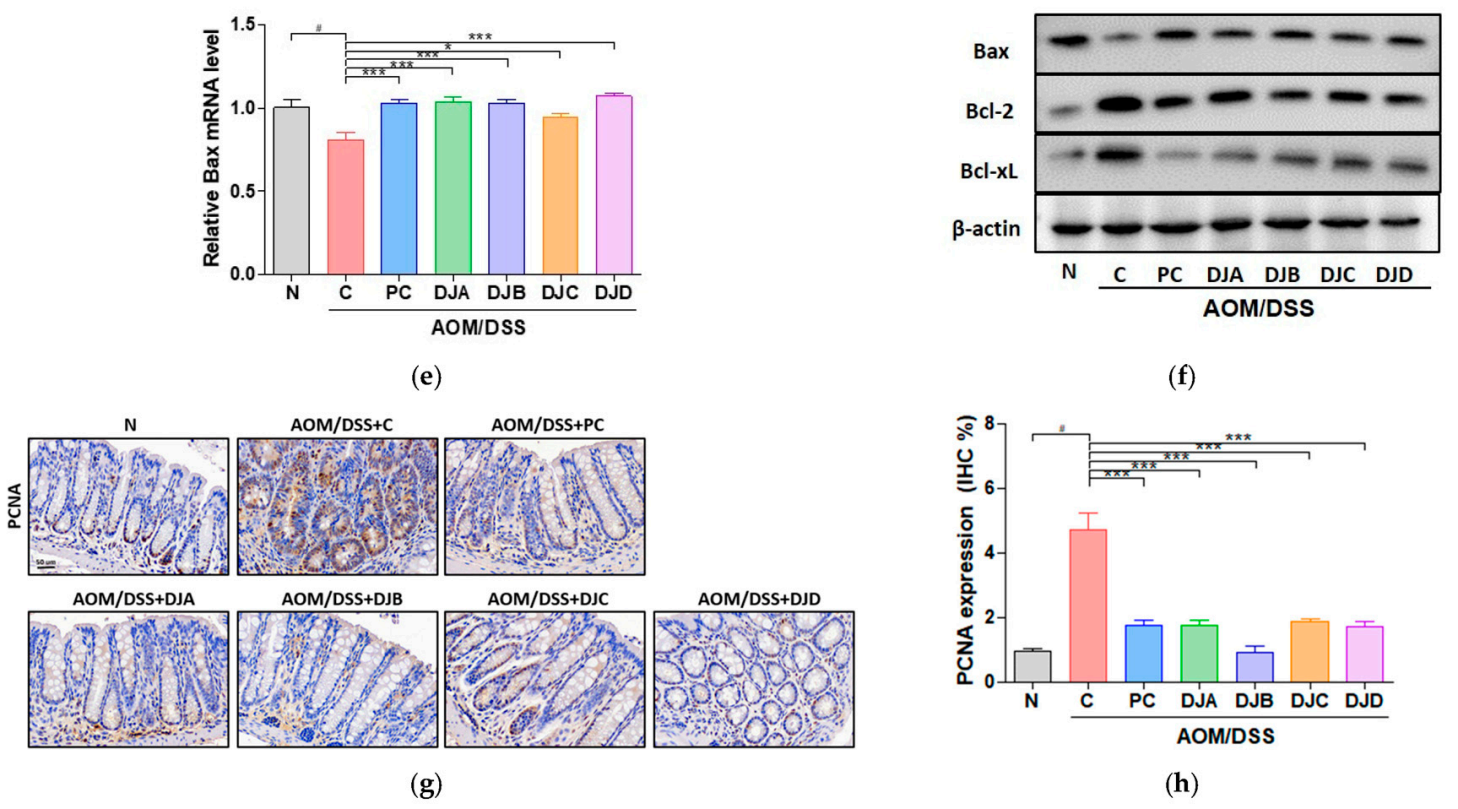
*Doenjang* suppresses tumor growth and promotes apoptosis in mice with AOM/DSS-induced CAC. (**a**) Tumor count and size in colon tissues (<2 mm or ≥2 mm). (**b**) Representative images showing tumor formation in colons from each group. Black dotted indicate tumor regions. (**c**–**e**) Relative transcript-level expression of *Bcl-2*, *Bcl-xL*, and *Bax* in colon tissues was measured by qRT-PCR. (**f**) Western blot analysis of apoptotic markers BCL-2, BCL-xL, and BAX in colon tissues. (**g**,**h**) Immunohistochemical detection of PCNA in colon tissue and corresponding IHC quantification. N, Normal control group; C, AOM/DSS group; PC, 75 mg/kg 5-ASA + AOM/DSS; DJA–DJD, *Doenjang* sample groups + AOM/DSS. # *p* < 0.05, compared to the N group; * *p* < 0.05, ** *p* < 0.01, and *** *p* < 0.001, compared to the C group.

To evaluate the effects of *Doenjang* on tumor development in CAC mice, tumor number and size were analyzed. The total number of tumors was significantly reduced in the PC group (6.6 ± 0.9) and *Doenjang*-treated groups, including DJA (7.4 ± 0.9), DJB (7.6 ± 0.9), DJC (7.0 ± 1.7), and DJD (7.2 ± 0.8), compared to the C group (10.2 ± 2.0) (Figure 3a). Similarly, the number of tumors larger than 2 mm was markedly lower in the PC (0.0 ± 0.0) and *Doenjang*-treated groups (DJA: 0.8 ± 0.4, DJB: 1 ± 1.2, DJC: 0.6 ± 0.5, DJD: 0.4 ± 0.9) than in the C group (3.0 ± 1.6). Representative images of colons from each group revealed fewer and smaller tumors in the 5-ASA and *Doenjang*-treated groups, further supporting the tumor-suppressive effects of *Doenjang* (Figure 3b).

To investigate the effects of *Doenjang* on apoptosis regulation, the transcript levels of apoptotic markers, including *Bcl-2*, *Bcl-xL*, and *Bax*, were measured in colon tissues. The C group showed significant upregulation of anti-apoptotic genes *Bcl-2* and *Bcl-xL* compared to the N group, indicating suppressed apoptosis. Treatment with 5-ASA and DJD significantly decreased the expression of these markers (Figure 3c,d). Conversely, the expression of the pro-apoptotic gene *Bax* was significantly reduced in the C group but was restored by 5-ASA and *Doenjang*, with significant increases observed in all groups (Figure 3e). Western blotting confirmed these results at the protein level, with elevated BCL-2 and BCL-xL expression and decreased BAX expression in the C group (Figure 3f). In contrast, treatment with 5-ASA and *Doenjang* samples reversed these changes, suggesting that *Doenjang* promotes apoptosis in CAC by modulating the apoptotic pathways.

Additionally, the expression of PCNA, a marker of cell proliferation, was analyzed through IHC (Figure 3g,h). The C group showed increased PCNA expression, indicating enhanced cell proliferation associated with tumor growth. In contrast, treatment with 5-ASA and *Doenjang* samples (DJA–DJD) significantly reduced PCNA expression, suggesting that *Doenjang* suppresses abnormal cell proliferation and may help inhibit tumor progression in CAC.

### 3.4. Doenjang Improves Intestinal Epithelial Barrier Integrity in Mice with AOM/DSS-Induced CAC

**Figure 4:**
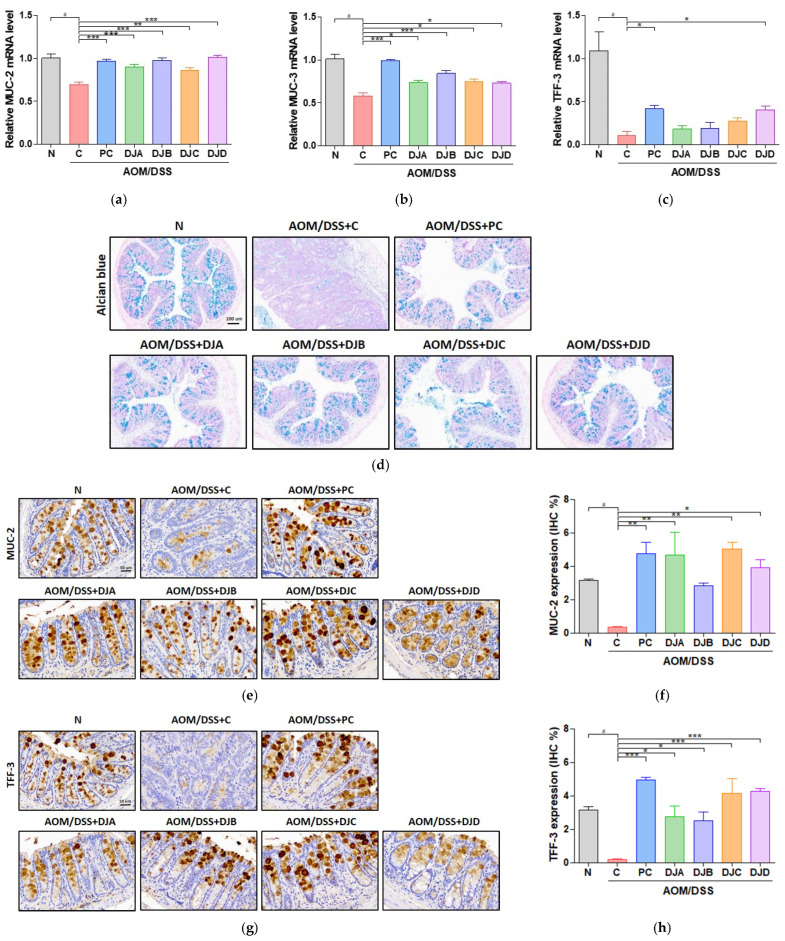
*Doenjang* improves the intestinal epithelial barrier integrity in mice with AOM/DSS-induced CAC. (**a**–**c**) Relative transcript-level expression of *MUC-2*, *MUC-3*, and *TFF-3* in colon tissues was measured by qRT-PCR. (**d**) Alcian blue staining to evaluate the mucus layer in colon tissues from each group. (**e**–**h**) Immunohistochemical detection of MUC-2 and TFF-3 in colon tissue and corresponding IHC quantification. N, Normal control group; C, AOM/DSS group; PC, 75 mg/kg 5-ASA + AOM/DSS; DJA–DJD, *Doenjang* sample groups + AOM/DSS. # *p* < 0.05, compared to the N group; * *p* < 0.05, ** *p* < 0.01, and *** *p* < 0.001, compared to the C group.

The effects of *Doenjang* on the intestinal epithelial barrier were evaluated by analyzing the expression of mucin-related genes and protective intestinal factors in the colon tissues of mice with AOM/DSS-induced CAC. The transcript-level expression of *MUC-2* and *MUC-3* was significantly reduced in the C group compared to the N group (Figure 4a,b). However, treatment with 5-ASA and *Doenjang* samples (DJA, DJB, DJC, DJD) significantly restored the expression of these genes in all *Doenjang*-treated groups. Similarly, expression of TFF-3, a key factor in mucosal repair, was significantly increased following treatment with 5-ASA and *Doenjang* (DJD group), indicating its role in promoting mucosal protection and repair (Figure 4c).

AB staining revealed a marked reduction in the mucus layer in the C group, indicating compromised barrier function. In contrast, treatment with 5-ASA and *Doenjang* samples restored the mucus layer across groups, suggesting improved intestinal barrier integrity (Figure 4d).

In addition, IHC for MUC-2 and TFF-3 revealed reduced expression in the C group, further confirming a weakened epithelial barrier. 5-ASA and *Doenjang* treatment increased the expression of both markers, indicating that *Doenjang* helps preserve intestinal barrier integrity by promoting mucus production and supporting mucosal protection (Figure 4e–h).

### 3.5. Doenjang Reduces Pro-Inflammatory Cytokines and Inflammatory Gene Expression via NF-κB Signaling Pathway in CAC Mice

**Figure 5:**
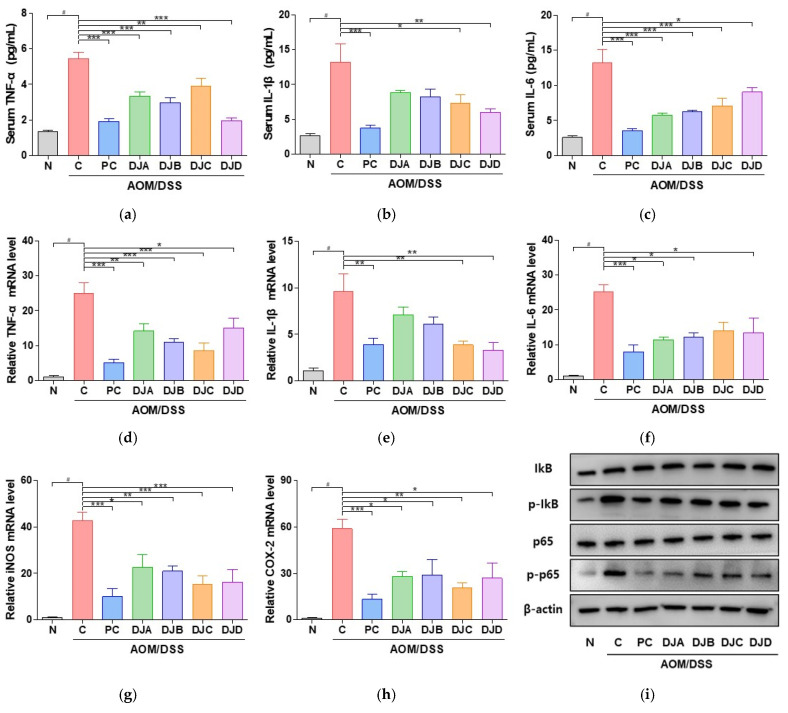
*Doenjang* attenuates inflammatory response through the NF-kB pathway in mice with AOM/DSS-induced CAC. (**a**–**c**) Serum levels of TNF-α, IL-1β, and IL-6 were measured by ELISA. (**d**–**h**) Relative transcript-level expression of TNF-α, IL-1β, IL-6, iNOS, and COX-2 in colon tissues was analyzed by qRT-PCR. (**i**) Western blot analysis of NF-κB pathway intermediaries in colon tissues. N, Normal control group; C, AOM/DSS group; PC, 75 mg/kg 5-ASA + AOM/DSS; DJA–DJD, *Doenjang* sample groups + AOM/DSS. # *p* < 0.05, compared to the N group; * *p* < 0.05, ** *p* < 0.01, and *** *p* < 0.001, compared to the C group.

The anti-inflammatory effects of *Doenjang* samples were evaluated by analyzing pro-inflammatory cytokine levels as well as inflammatory gene expression in mouse models of AOM/DSS-induced CAC. The serum levels of TNF-α, IL-1β, and IL-6 were significantly elevated in the C group compared to those in the N group (Figure 5a–c). However, treatment with *Doenjang* samples led to a significant reduction in these cytokine levels, indicating a suppressive effect on inflammation. At the mRNA level, TNF-α, IL-1β, IL-6, iNOS, and COX-2 were markedly upregulated in the C group (Figure 5d–h). In contrast, these inflammatory markers was significantly downregulated in the *Doenjang*-treated groups, demonstrating the potential of *Doenjang* to modulate inflammatory gene expression.

Western blotting was performed to assess the activation of the NF-κB pathway, a key regulator of inflammatory responses (Figure 5i). The C group exhibited increased phosphorylation of p65 and IκB, indicating activation of the NF-κB pathway. However, treatment with *Doenjang* samples inhibited the phosphorylation of both p65 and IκB, thereby suppressing NF-κB activation. These results suggest that *Doenjang* exerts its anti-inflammatory effects by downregulating the NF-κB pathway, thereby reducing the expression of pro-inflammatory cytokines and inflammatory mediators at both the gene and protein levels. This indicates that *Doenjang* may play a protective role in AOM/DSS-induced CAC through its anti-inflammatory properties.

### 3.6. Doenjang Modulates Gut Microbiota Composition and Diversity in CAC Mice

**Figure 6:**
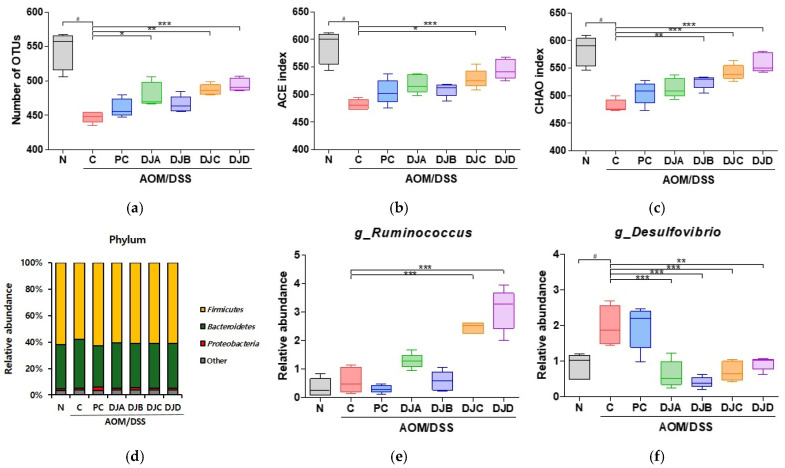
*Doenjang* modulates gut microbiota composition and diversity in mice with AOM/DSS-induced CAC. (**a**–**c**) α-Diversity indices, including the number of OTUs, ACE index, and Chao1 index were analyzed to assess microbial richness. (**d**) Phylum-level composition of gut microbiota in each group. (**e**,**f**) Relative abundance of *Ruminococcus* and *Desulfovibrio* at the genus level in colon tissues. N, Normal control group; C, AOM/DSS group; PC, 75 mg/kg 5-ASA + AOM/DSS; DJA–DJD, *Doenjang* sample groups + AOM/DSS. # *p* < 0.05, compared to the N group; * *p* < 0.05, ** *p* < 0.01, and *** *p* < 0.001, compared to the C group.

To evaluate the effect of *Doenjang* on gut microbiota composition in mice with AOM/DSS-induced CAC, fecal samples were analyzed by NGS, and α-diversity indices and microbial community profiles were analyzed. The ACE index, Chao1 index, and number of OTUs were significantly decreased in the C group compared to the N group, indicating reduced microbial richness due to CAC progression. However, treatment with 5-ASA and *Doenjang* samples (DJA–DJD) significantly restored these indices, suggesting that *Doenjang* treatment modulates gut microbial diversity (Figure 6a–c; Table 4). At the phylum level, the dominant microbial phyla included Proteobacteria, Firmicutes, and Bacteroidetes across all groups (Figure 6d). At the genus level, the relative abundance of *Ruminococcus*, a beneficial gut bacterium involved in intestinal health, was increased in the *Doenjang*-treated groups compared to the C group, suggesting a positive shift in gut microbial composition (Figure 6e). Conversely, the relative abundance of *Desulfovibrio*, a harmful bacterium linked to inflammation, was significantly elevated in the C group. Treatment with 5-ASA and *Doenjang* samples markedly reduced *Desulfovibrio* levels (Figure 6f).

## 4. Discussion

Recent studies have reported that *Doenjang* has various health benefits, including anti-inflammatory, antioxidant, anti-obesity, anti-hypertensive, and anticancer properties [22,23,24]. Traditional and commercial *Doenjang* differ fundamentally in production approaches—traditional products rely on spontaneous, long-term fermentation guided by region-specific microbial ecologies, whereas commercial products generally employ defined starter cultures and controlled conditions—leading to distinct microbial communities [18,25,26]. However, safety remains a critical consideration, as BAs may accumulate depending on microbial membership, substrate availability, and process control [27,28]. Despite the recognition that manufacturing practices influence both the microbiome and the chemical contents of *Doenjang*, an integrated evaluation linking manufacturing-dependent microbiota, BA levels, and in vivo functional outcomes has remained limited. Accordingly, this study aimed to analyze the microbial communities and BA content in four different *Doenjang*, and to compare and evaluate their anticancer effects in mouse models of AOM/DSS-induced CAC.

Bacterial composition analysis of *Doenjang* samples revealed that all samples were dominated by Firmicutes, Bacilli, and Bacillales at the phylum, class, and order levels, respectively (Figure 1a–c). However, the bacterial communities were more diverse at finer taxonomic levels (family, genus, and species) (Figure 1d–f). At the species level, *Bacillus licheniformis*, *Bacillus subtilis*, *Tetragenococcus halophilus*, and *Staphylococcus aureus* were observed. Studies have shown that *Doenjang* harbors dominant bacterial species, such as *Bacillus licheniformis*, *Bacillus subtilis*, and *Tetragenococcus halophilus* [18,29], which contribute to the composition of gut microbiota, helping beneficial effects such as anti-inflammation, improvement in abdominal bloating [30,31,32]. *Bacillus licheniformis* and *Bacillus subtilis* are not known to have toxic genes, whereas the *Tetragenococcus halophilus* strain isolated from *Doenjang* exhibited resistance to ciprofloxacin [33,34,35]. Accordingly, further safety assessment studies are required, particularly through the isolation and characterization of *Tetragenococcus halophilus* strains present in the *Doenjang* used in this study.

BAs are low-molecular-weight organic bases that are primarily formed through microbial decarboxylation of amino acids during fermentation processes. Among the various BAs commonly detected in fermented foods, histamine, tyramine, putrescine, and cadaverine occur with the highest frequency, with histamine and tyramine being considered the most toxic [36]. The generally recommend maximum levels in food products are 100 mg/kg for histamine and 100–800 mg/kg for tyramine, and excessive intake of BAs is associated with various adverse health effects [37]. Soy-based fermented foods are known to contain significantly higher levels of BAs compared to other fermented products such as fish, meat, or dairy [37]. Notably, *Doenjang* has been reported to contain histamine levels up to 2795 mg/kg and tyramine levels up to 6616 mg/kg [38]. However, due to the small serving sizes typically consumed, actual intake may remain below toxicologically significant thresholds for most individuals. To evaluate the safety of BAs in *Doenjang*, we measured BA content in samples used in this study. The histamine content of all *Doenjang* ranged from 141.9 to 928.61, and the tyramine content ranged from 186.59 to 888.86, confirming that the BA content varied depending on the production method. When *Doenjang* was administered orally for 8 weeks, and the side effects of the BA content in samples were analyzed, no noticeable side effects, including weight changes or abnormal behavior, such as reduced locomotor activity, were observed (Figure 2a,b).

Next, we found that *Doenjang* effectively mitigates CAC progression in mice with AOM/DSS-induced CAC by targeting key inflammatory and apoptotic pathways. In AOM/DSS-induced CAC mouse model, treatment with four *Doenjang* samples was associated with reduced tumor formation, attenuation of intestinal inflammation, and improvement in intestinal epithelial integrity. The anti-inflammatory effects of *Doenjang* were evident from its ability to suppress pro-inflammatory cytokines, such as TNF-α, IL-1β, and IL-6, which play pivotal roles in the initiation and progression of CRC. Chronic activation of the NF-κB signaling pathway is a well-known driver of inflammation and tumor development in the colon [39]. Our study showed that all *Doenjang* samples inhibited the phosphorylation of p65 and IκB, key components of the NF-κB pathway, thereby reducing pro-inflammatory cytokine levels and attenuating inflammatory gene expression. These results suggest that the anti-inflammatory properties of *Doenjang* are closely linked to its ability to regulate NF-κB signaling, a critical pathway involved in inflammation-driven carcinogenesis. In addition to its anti-inflammatory effects, *Doenjang* was observed to modulate apoptotic markers, which may be relevant to the regulation of cell proliferation in this mouse model of AOM/DSS-induced CAC. The balance between pro-apoptotic and anti-apoptotic proteins determines cell fate, such as cancer development [40]. In our study, we observed that *Doenjang* induced apoptosis by decreasing the expression of anti-apoptotic markers, such as *Bcl-2* and *Bcl-xL*, while increasing the expression of pro-apoptotic marker *Bax* in colon tissue. These findings indicate that *Doenjang* exerts its anticancer effects not only by reducing inflammation but also by inducing apoptosis in tumor cells, contributing to the suppression of CRC progression.

The modulation of gut microbiota composition by *Doenjang* played a crucial role in improving intestinal homeostasis. The gut microbiota is a key regulator of intestinal health, and its imbalance (dysbiosis) is associated with chronic inflammation and CRC progression [41]. Our gut microbiota analysis revealed that *Doenjang* increased the diversity of gut microbial communities and enhanced the abundance of beneficial bacteria, such as *Ruminococcus*, which are known to play a critical role in maintaining intestinal barrier integrity and reducing inflammation, as reported in previous studies [42]. Conversely, the harmful bacterium *Desulfovibrio*, often linked to inflammation and CRC through its production of hydrogen sulfide (H_2_S), was significantly reduced in the *Doenjang*-treated groups [43,44]. Similarly, a previous study reported that fermented soy food (*Miso*) intake modulated gut microbiota by increasing beneficial bacteria while reducing *Desulfovibrio* abundance and inflammatory markers, further supporting its role in gut health regulation [45]. Overall, the observed changes suggest that *Doenjang* supports gut health by fostering a favorable microbial environment, potentially contributing to CRC prevention. However, as *Doenjang* contains both probiotic bacteria and various bioactive compounds, further studies are needed to identify the specific components responsible for its anti-cancer effects.

## 5. Conclusions

This study compared the microbial communities and BA contents of *Doenjang* produced across multiple Korean regions and evaluated anticancer efficacy in a mouse model of AOM/DSS-induced CAC. At the species level, the microbial communities differed among the four treatment groups, but overall, probiotic taxa predominated, and BA contents varied among samples. Despite these differences, all *Doenjang* samples ameliorated CAC features, such as colon length and colon weight-to-length ratio. Mechanistically, the samples suppressed tumor growth via apoptosis regulation; restored epithelial integrity by modulating MUC-2, MUC-3, and TFF-3 expression; and attenuated inflammatory cytokine expression through NF-κB pathway modulation. Furthermore, administration of *Doenjang* increased beneficial bacteria abundance and enhanced gut microbiota diversity. Taken together, these findings suggest that *Doenjang* has the potential to serve as a safe and effective dietary intervention for the prevention and management of CRC.

## Figures and Tables

**Table 1:** Information on *Doenjang* samples.

	Methods	Production Region	Sodium (mg/100 g)	Biogenic Amines	
				Histamine (mg/kg)	Tyramine (mg/kg)
DJA (*Doenjang* A)	Traditional	Sunchang, Jeonbuk	4072.53	141.90	284.27
DJB (*Doenjang* B)	Traditional	Sancheong, Gyeongnam	3743.47	151.13	886.86
DJC (*Doenjang* C)	Traditional	Seongju, Gyeongbuk	4364.04	794.86	215.89
DJD (*Doenjang* D)	Industrial	Sunchang, Jeonbuk	4028.21	928.61	186.59

**Table 2:** The dosing of each group in mice.

Group	Treatment	Sample
N	Saline(i.p.)/Autoclaved water	Autoclaved water
AOM/DSS + C	AOM (i.p., 10 mg/kg)/2% DSS (drinking water)	Autoclaved water
AOM/DSS + PC	AOM (i.p., 10 mg/kg)/2% DSS (drinking water)	5-ASA (75 mg/kg)
AOM/DSS + DJA	AOM (i.p., 10 mg/kg)/2% DSS (drinking water)	DJA (200 mg/kg)
AOM/DSS + DJB	AOM (i.p., 10 mg/kg)/2% DSS (drinking water)	DJB (200 mg/kg)
AOM/DSS + DJC	AOM (i.p., 10 mg/kg)/2% DSS (drinking water)	DJC (200 mg/kg)
AOM/DSS + DJD	AOM (i.p., 10 mg/kg)/2% DSS (drinking water)	DJD (200 mg/kg)

Note: Normal control group (N), negative control group (AOM/DSS + C), positive control group (AOM/DSS + PC), and four *Doenjang* sample dose groups (AOM/DSS + DJA, AOM/DSS + DJB, AOM/DSS + DJC, AOM/DSS + DJD).

**Table 3:** Primer Sequences.

Gene	Forward (5′-3′)	Reverse (5′-3′)
*Bax*	AGACAGGGGCCTTTTTGCTAC	AATTCGCCGGAGACACTCG
*Bcl-2*	GCTACCGTCGTGACTTCGC	CCCCACCGAACTCAAAGAAGG
*Bcl-X_L_*	GGCACTGTGCGTGGAAAGCGTA	CCGCCGTTCTCCTGGATCCA
*TNF-α*	CTGAACTTCGGGGTGATCGG	GGCTTGTCACTCGAATTTTGAGA
*IL-1β*	CAACCAACAAGTGATATTCTCCATG	GATCCACACTCTCCAGCTGCA
*IL-6*	TGTCTATACCACTTCACAAGTCGGAG	GCACAACTCTTTTCTCATTTCCAC
*COX-2*	TTTGGTCTGGTGCCTGGTC	CTGCTGGTTTGGAATAGTTGCTC
*iNOS*	CGAAACGCTTCACTTCCAA	TGAGCCTATATTGCTGTGGCT
*MUC-2*	ATGCCCACCTCCTCAAAGAC	GTAGTTTCCGTTGGAACAGTGAA
*MUC-3*	CGTGGTCAACTGCGAGAATGG	CGGCTCTATCTACGCTCTC
*TFF-3*	TAATGCTGTTGGTGGTCCTG	CAGCCACGGTTGTTACACTG
*β-actin*	CGGTTCCGATGCCCTGAGGCTCTT	CGTCACACTTCATGATGGAATTGA

**Table 4:** Alpha diversity index values.

Group	Number of OTUs	ACE Index	Chao 1 Index
N	544.0 ± 26.8	586.1 ± 29.6	581.2 ± 26.2
AOM/DSS + C	447.2 ± 7.9 ^#^	482.3 ± 9.0 ^#^	481.6 ± 11.1 ^#^
AOM/DSS + PC	460.8 ± 13.2	505.3 ± 22.5	505.0 ± 20.3
AOM/DSS + DJA	480.2 ± 17.2 *	519.2 ± 16.4	514.0 ± 17.2
AOM/DSS + DJB	466.2 ±11.8	508.9 ± 12.4	524.7 ± 11.6 **
AOM/DSS + DJC	487.2 ± 7.7 **	528.2 ± 16.8 *	542.3 ± 14.0 ***
AOM/DSS + DJD	494.4 ± 8.8 ***	545.8 ± 18.1 ***	559.4 ± 17.7 ***

Note: Values are expressed as mean ± standard deviation (SD). Statistical significance was determined by one-way ANOVA followed by Tukey’s multiple comparison test. ^#^ *p* < 0.001 compared with N group. * *p* < 0.05, ** *p* < 0.01, *** *p* < 0.001 compared with AOM/DSS + C group.

## Data Availability

The original contributions presented in this study are included in the article. Further inquiries can be directed to the corresponding author.
